# On the Origin of *Pantepui montane biotas*: A Perspective Based on the Phylogeny of *Aulacorhynchus* toucanets

**DOI:** 10.1371/journal.pone.0067321

**Published:** 2013-06-26

**Authors:** Elisa Bonaccorso, Juan M. Guayasamin

**Affiliations:** 1 Centro de Investigación en Biodiversidad y Cambio Climático, Universidad Tecnológica Indoamérica, Quito, Pichincha, Ecuador; 2 Biodiversity Institute, University of Kansas, Lawrence, Kansas, United States of America; Institut de Biologia Evolutiva - Universitat Pompeu Fabra, Spain

## Abstract

To understand the origin of Pantepui montane biotas, we studied the biogeography of toucanets in the genus *Aulacorhynchus*. These birds are ideal for analyzing historical relationships among Neotropical montane regions, given their geographic distribution from Mexico south to Bolivia, including northern Venezuela (Cordillera de la Costa), and the Pantepui. Analyses were based on molecular phylogenies using mitochondrial and nuclear DNA sequences. Topology tests were applied to compare alternative hypotheses that may explain the current distribution of *Aulacorhynchus* toucanets, in the context of previous hypotheses of the origin of Pantepui montane biotas. Biogeographic reconstructions in RASP and Lagrange were used to estimate the ancestral area of the genus, and an analysis in BEAST was used to estimate a time framework for its diversification. A sister relationship between the Pantepui and Andes+Cordillera de la Costa was significantly more likely than topologies indicating other hypothesis for the origin of Pantepui populations. The Andes was inferred as the ancestral area for *Aulacorhynchus*, and the group has diversified since the late Miocene. The biogeographic patterns found herein, in which the Andes are the source for biotas of other regions, are consistent with those found for flowerpiercers and tanagers, and do not support the hypothesis of the geologically old Pantepui as a source of Neotropical montain diversity. Based on the high potential for cryptic speciation and isolation of Pantepui populations, we consider that phylogenetic studies of additional taxa are important from a conservation perspective.

## Introduction

Given their old geological origin, geographic isolation, and endemic biota, the highlands of the Guianan Shield have been a source of inspiration for explorers and naturalists alike. This biogeographic region, known as ‘Pantepui’ [1], [2], is formed by mountains derived from the Precambrian sandstone rocks of the Roraima Group in southern Venezuela, western Guyana, and northern Brazil [3]. Since the Jurassic–Cretaceous period, progressive erosion of the Roraima sandstone has shaped the landscape, leaving behind spectacular tabletop mountains (*tepuis*) surrounded by savannas and tropical forests [4].

The ancient age of the Pantepui has long been associated with the notion of undisturbed and continuous processes of biological diversification and isolation [5], [6]. This idea, known as the Plateau Theory, proposes that the ‘*Pantepui fauna is the remnant of a fauna formerly widespread on a plateau now dissected by erosion into separate tepuis’* [1]. Initially, this hypothesis was supported by remarkable levels of plant and animal endemism [7], [8]. However, thorough analysis of plant distributions revealed that endemism was lower than previously estimated [9]. Also, topographic studies showed that most tepuis are effectively connected to each other and to lowland ecosystems in the present [5] and were likely connected in the past [10]. In fact, palaeoecological studies based on Quaternary sediments indicate vertical displacement of vegetation in the middle and lower elevations of these table mountains [6]. These results do not deny the plausibility of the Plateau Theory as an explanation for the origin of some Pantepui endemics, but show that evolution in isolated tepuis might not be the generality for all such organisms.

Interestingly, many Pantepui animals and plants are neither closely related to those of the surrounding lowlands, nor endemic to particular tepuis, but have taxonomic affinities to populations found in other Neotropical montane regions [1], [10], [11], [12], [13]. Chapman [14] concluded that, although a quarter of the distinctive birdlife of mounts Roraima and Duida derived from tropical elements of the surrounding Amazonian habitats, about half of the species might have origins among the Andean tropical and subtropical avifaunas. Historical relationships between these avifaunas were established by the sharing of species within genera, subspecies within species, or even the same subspecies.

Regarding the origin of biogeographic affinities between Pantepui and Andean species, Chapman [14] argued that this pattern *‘may be explained by the disappearance of their common ancestor, or connecting forms, in the intervening area’*, and that *‘the cause of their disappearance is attributed chiefly to the influence of climatic changes.’* Tate [15] was more explicit about the mechanism of such a scenario. He proposed that during a time of lower temperatures (such as the Last Glacial Maximum), lower mountain ranges may have served as bridges of suitable habitat that allowed biotic exchange from the Andes to the Pantepui and *vice versa*. Following Chapman’s argument, common ancestors or connecting forms that lived in these bridges would have disappeared with increasing temperatures.

An alternate hypothesis, proposed by Mayr and Phelps [1], states that the subtropical avifauna of Pantepui is derived from that of the Andes and other subtropical regions by ‘island hopping,’ not only during the Quaternary period but in multiple occasions in the past. Here, the flexibility of the time premise is based on the idea that animals have different dispersal abilities that may be expressed depending on external or internal stimuli. Whereas some species may disperse with changing conditions in the environment (e.g., lowering of life zones during glaciations), others may disperse in response to the evolution of morphological, physiological, and behavioral traits that facilitate dispersal [16]. Haffer [17] supported the idea of ‘island hopping,’ but restricted the time-window for dispersal events to the Pleistocene.

Croizat [18] opposed the idea of dispersal between the Andes and the Pantepui, but suggested that, if any chance of dispersal existed, the Pantepui should be the source of migrants, given its ancient age. A similar proposition based on the old age of the tepuis was applied to propose a Pantepuian origin for Andean members of the *Diglossa carbonaria* and *D. lafresnayi* species groups [19]. More recently, Givnish et al. [20], [21] identified the Guianan Shield as the center of origin for bromeliads, which dispersed subsequently to the Brazilian Shield, the Andes, the Amazon Basin, and Central America, and re-colonized the Guianan Shield. Bromeliads arose ∼100 million years ago (Mya) [21], when the Guianan Shield was still experiencing erosion (up to70 Mya; [22]) and before significant uplift of the Andes occurred in the Miocene–Pliocene [23], [24].

In summary, current hypotheses for the origin of Pantepui biotas with close affinities to other montane regions state that: (1) they derived from taxa with broad distributions in the Neotropical montane regions that differentiated (via vicariance) into the Pantepui regional biota, sometime in the past [14], [25] including the time after the Pleistocene [15]; (2) they have their origin in multiple dispersal events from the Andes and other montane regions at different points in time [1], including the Pleistocene [17]; and (3) they have derived from an ‘ancient tepui’ biota that has contributed elements to younger montane regions, including the Andes [18], [19], [21]. Neither parsimony analysis of endemicity based on distributions of >400 Neotropical montane species [12], nor comparisons of area cladograms of 43 co-distributed species complexes [13], have been able distinguish among these hypotheses. Although a plethora of comparative studies will be needed to answer these questions, only very limited phylogenetic information is currently available [26], [20], [21], [27], [28].

**Figure 1:**
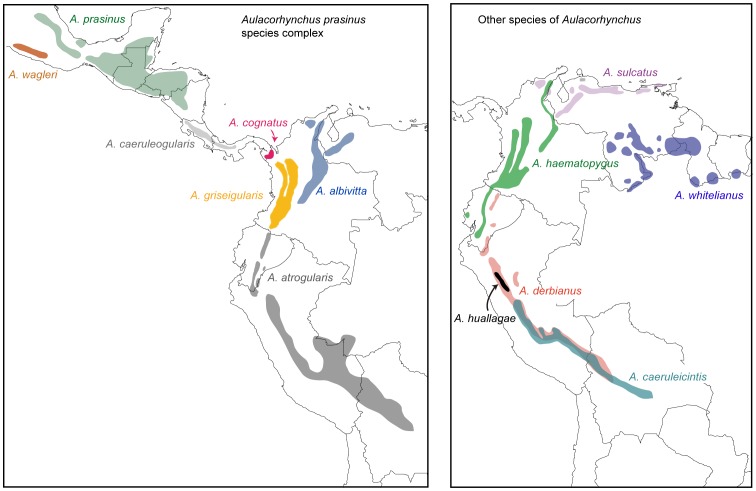
Geographic distribution of species in *Aulacorhynchus*. Subspecies in the *Aulacorhynchus prasinus* complex are shown as independent evolutionary units following Puebla-Olivares et al. [31] and Bonaccorso et al. [32]; taxonomy of South American species, other than *A. prasinus*, follows Remsen et al. [29].

To understand biological endemism in the Pantepui highlands, we studied the phylogeny of toucanets in the genus *Aulacorhynchus*. These toucanets are ideal for understanding historical relationships among Neotropical montane regions because they inhabit subtropical and temperate forests from Mexico south to Bolivia in a fragmented distribution (Figure 1). Former taxonomy [29] recognized six species: *A. prasinus*, a species complex [30], [31], that is widely distributed across the Mesoamerican highlands and the Andes; *A. coeruleicinctis* and *A. huallagae* in the Central Andes; *A. haematopygus* in the Northern Andes; *A. sulcatus* in the mountains of northern Venezuela (Cordillera de la Costa), west to the Venezuelan Andes and northeastern Colombia; and, most relevant, *A. derbianus* in the Andes from Bolivia to northern Ecuador, and discontinuously in the Pantepui. More recently, a phylogenetic study by Bonaccorso et al. [32] revealed that *A. derbianus* is paraphyletic: Andean populations of this species (*A. d. derbianus*) are more closely related to *A. sulcatus*, than to the Pantepui populations (*A. d. duidae*, *A. d. whitelianus*, and *A. d. osgoodi*). Based on this work, a new taxonomy consistent with the evolutionary history of the group was proposed, where Andean populations of *A. derbianus* retain the species name and the Pantepui populations were designed as *A. whitelianus* [32], [29].

Herein, we assess historical hypotheses for the origin of Pantepui *Aulacorhynchus* using molecular evidence generated by Bonaccorso et al. [32]. Specifically, we aim to (1) test the best estimate of phylogeny against topologies congruent with other hypotheses for the origin of the Pantepui species, (2) reconstruct the biogeographic history of the group and its pattern of colonization across the Neotropics, and (3) place the diversification of *Aulacorhynchus* in a temporal framework. We present our results in the context of previous hypotheses regarding the origin of Pantepui biotas to understand patterns of distribution across Neotropical montane regions.

## Materials and Methods

### Taxon and Gene Sampling

Genetic sequence data are available from GenBank (accession numbers AY959855, AY959828 [33], and JF424372–JF424596 [32]). Hypothesis testing and biogeographic reconstructions were performed on phylogenetic trees estimated using 34 samples that cover all species and subspecies of South American *Aulacorhynchus*, and includes Mesoamerican and South American representatives of the *A. prasinus* species group (Table S1). To polarize character states, we included samples from all other toucan genera (*Andigena*, *Selenidera*, *Pteroglossus*, and *Ramphastos*) as outgroups.

Molecular character sampling consisted of the mitochondrial genes NADH Dehydrogenase Subunit 2 (ND2) and cytochrome b (cyt*b*), and the nuclear genes β-Fibrinogen intron 7 (βfb7) and Transforming Growth Factor beta 2 intron 5 (TGFβ2.5). Because DNA from *Aulacorhynchus huallagae* was obtained from dry toe pads that resulted in low yields, only ND2 sequences were available for analyses. Also, nuclear sequences of *Andigena hypoglauca* were concatenated with mitochondrial sequences of *Andigena cucullata* published by Weckstein [33]. These combined sequences are technically chimeric, however, their appropriateness to root the trees is justified because sequences from both taxa are, in all probability, more closely related to one another than to those of other species in the combined dataset (i.e., we assumed that *Andigena* is monophyletic). This later affirmation was supported by a phylogenetic analysis of cytb sequences for species of all Ramphastidae available on GenBank (results not shown). With the aforementioned exceptions, all individuals were represented by the four loci.

A hypothetical temporal frame for the diversification of *Aulacorhynchus* was generated using a broader sampling of 63 individuals sequenced for cyt*b* (Table S1). Laboratory procedures for obtaining all sequences are detailed elsewhere [32], [33].

### Phylogeny

Best-fit models of evolution were estimated for each gene in jModeltest [34] under the Akaike Information Criterion. These preliminary analyses indicated the following best-fit models: TIM+I+Г, for ND2; TrN+I+Г, for cyt*b*; HKY+Г, for βfb7; and GTR+I, for TGFβ2.5. Maximum likelihood trees were obtained in GARLI (ver. 2.0; [35]), to allow phylogeny estimation using data partitions. We implemented a partition by gene, assigning each gene its best-fit model ‘family.’ Individual solutions were selected after 5000 generations with no significant improvement in likelihood (significant topological improvement set at 0.01). Final solutions were selected when the total improvement in likelihood score was <0.05, using default values for the remaining GARLI settings [35]. Ten independent runs were completed to assure consistency of likelihood scores.

Bayesian trees were obtained in MrBayes 3.2 [36], also partitioning data by gene. Model parameters were unlinked between partitions, except topology and branch lengths. Analyses consisted of two independent runs of 10×10^6^ generations and four Markov chains (temperature = 0.20), with trees sampled every 1000 generations. Of the 10,000 trees resulting, the first 2500 were discarded as burn-in. The remaining trees were combined to calculate the posterior probabilities in a 50% majority-rule consensus tree.

### Hypothesis Testing

**Figure 2:**
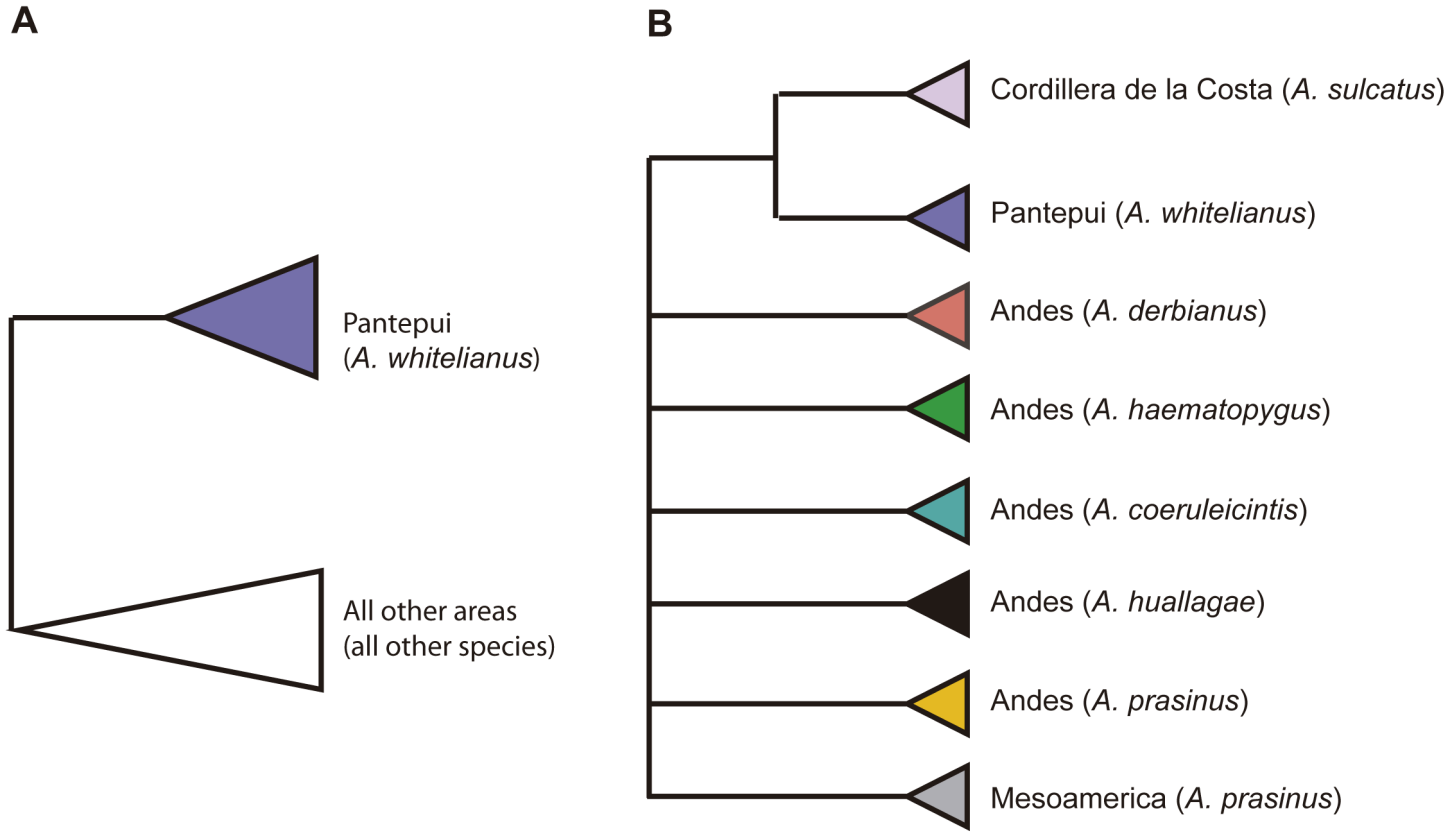
Alternative hypotheses for relationships among biogeographic areas occupied by species in *Aulacorhynchus*. A: Topology congruent with the ‘ancient tepui’ hypothesis, showing a sister relationship between Pantepui taxa and a clade uniting taxa from all other Neotropical montane regions. B: Topology congruent with a hypothesis where species from Pantepui and Cordillera de la Costa are more closely related to each other than to species from other regions.

The best estimate of phylogeny, in which *Aulacorhynchus whitelianus* (Pantepui) is sister to *A. sulcatus*+*A. derbianus* (Cordillera de la Costa+Andes), was tested against two other topologies depicting alternate relationships between the Pantepui lineage and other *Aulacorhynchus* lineages. The first alternate hypothesis was based on the idea of ancient origins of Pantepui endemics (‘ancient tepui’ hypothesis), with a sister relationship between Pantepui taxa and a clade uniting taxa from all other Neotropical montane regions (Figure 2A). This possibility was represented by a topology in which *A. whitelianus* was sister to all other *Aulacorhynchus.* If that relationship were to hold, additional analyses should be used to test among Patepui origin and posterior dispersal, Andean origin and posterior dispersal, or vicariance. The second hypothesis was based on the findings of a previous phylogenetic study [26], in which the Pantepui lineage of *Myioborus* redstarts was sister to a lineage from the Cordillera de la Costa in northern Venezuela. This sister relationship between Pantepui and Cordillera de la Costa was represented by a tree in which *A. whitelianus* was placed as sister to *A. sulcatus*, the only *Aulacorhynchus* in the Cordillera de la Costa (Figure 2B).

We applied a Bayesian approach to hypothesis testing. This procedure consisted of taking the post-burn-in trees from the posterior probability distribution and filtering to detect trees compatible with the alternate hypothesis in PAUP. The number of the trees retained estimates the posterior probability that the hypothesis is correct [37].

### Reconstruction of Ancestral Areas

To infer the biogeographic history of *Aulacorhynchus*, we followed two approaches: Dispersal Vicariance Analysis (DIVA) in a Bayesian framework, implemented in RASP (ver 1.107; [38]), and a Dispersal-Extinction-Cladogenesis model (DEC) developed for a likelihood framework, in Lagrange (ver. 20120508; [39]). Both methods allow estimating the probability of optimizing ancestral areas at each node of a phylogenetic tree. In RASP, the Bayesian binary MCMC analysis takes into account the inherent uncertainty of the phylogenetic inference by optimizing ancestral areas over multiple trees [40]. In Lagrange, the DEC model specifies a instantaneous transition rate matrix between ranges along branches of a phylogenetic tree, and applies it to estimating likelihoods of ancestral states [39].

Geographic regions analyzed, included: Northern Andes (from the Huancabamba Depression north to western Venezuela), Central Andes (from the Huancabamba Depression south to Bolivia), Cordillera de la Costa, Pantepui, and Mesoamerica. The segregation among geographic regions takes into account the existence of major barriers that may limit dispersal and gene flow [13] and separate the geographic ranges of some populations or species in *Aulacorhynchus*. Also, treatment of the Northern and Central Andes as different biogeographic units takes into consideration phylogenetic studies suggesting that patterns of geographic diversification in the region may proceed in a south-to-north direction, matching the sequential uplift of the Andes [41], [42].

Areas were assigned to species and populations, as follows: (1) Central Andes–*Aulacorhynchus prasinus* from South America, *A. derbianus*, *A. coeruleicinctis*, *A. huallagae*; (2) Northern Andes–*Aulacorhynchus prasinus* from South America, *A. derbianus, A. haematopygus, A. sulcatus* from the Andes of Venezuela; (3) Cordillera de la Costa–*A. sulcatus* from Cordillera de la Costa; (4) Pantepui–*A. whitelianus*; and (5) Mesoamerica–*Aulacorhynchus prasinus* from Mesoamerica. Minor ridges such as Sierra de Perijá (between northern Colombia and Venezuela) and Sierra Nevada de Santa Marta (northeastern Colombia) were considered as part of the Northern Andes, to limit the number of areas included in analyses. Guided by current species distributions, we restricted the number of maximum areas to two, in both (RASP and Lagrange) analyses.

Bayesian binary MCMC (RASP) and DEC (Lagrange) analyses were run based on a set of Bayesian trees obtained using the Bayesian Markov chain Monte Carlo (MCMC) procedure implemented in BEAST (v.1.6.2; [43]). In this analysis, outgroups were excluded and species in *Aulacorhynchus* were constrained to be monophyletic. The justification behind this procedure was that species monophyly was supported by Bayesian posterior probabilities >0.95 in the previous phylogenetic analyses run over the same dataset (see Phylogeny, above). BEAST was run with a relaxed-clock model in which each gene was assigned its best-fit model of evolution, uncorrelated rates at each branch (estimated from a log-normal distribution), and a Yule speciation process tree prior. The program was run for 10×10^6^ generations, sampling every 1000 generations. Convergence of chains to stationary was confirmed by inspection of posterior density of parameters in Tracer [44]; topological convergence was inspected in AWTY [45]. After discarding 2000 trees as burn-in, a maximum clade probability tree was obtained using the remaining 8000 trees.

Bayesian binary MCMC analysis in RASP was performed over the 8000 post-burnin BEAST trees using the BEAST maximum clade probability tree as a ‘condense tree’. The analysis was conducted by setting the evolutionary model to F81+ G, running it for 50,000 cycles using 10 chains, sampling every 100 cycles and discarding 100 trees, and setting root distribution to null.

Likelihood DEC analyses in Lagrange were performed over the maximum clade credibility tree obtained in BEAST. In range constraints', adjacency of areas was allowed only between areas that were geographically contiguous (i.e., between the Northern Andes and all other areas, and between Cordillera de la Costa and Pantepui). Maximum range size was set to two areas, and ranges allowed in the analysis included all possible combinations within those imposed by adjacency and maximum range size. Dispersal was only allowed between adjacent areas. Baseline rates of dispersal and local extinction were estimated by the program.

### Hypothetical Time of Diversification in *Aulacorhynchus*

To estimate an approximate time frame for the speciation of *Aulacorhynchus* toucanets, we applied a second BEAST analysis over the cyt*b* mitochondrial gene dataset. We used a mean substitution rate guided by previous estimates for the avian genome (0.008–0.0095 Ma^–1^, for cyt*b* [46] and ∼0.0105 for an average rate among birds [47]). The prior value for the Euclidean mean was set at 0.0105 Ma^–1^ and the prior standard variation of this parameter was set at 0.0034; both values were allowed to vary between zero and infinity. Because applying such general calibration disregards rate heterogeneity of the mitochondrial molecular clock across avian lineages [48], [49], this analysis was not intended to produce precise estimations of diversification times. Instead, we present the results of the analysis as a first approximation that should be tested against independent sources of temporal evidence when they become available.

Analyses were run using the GTR+Г+I model under a relaxed clock. Uncorrelated rates at each branch were estimated from a log-normal distribution. Species in *Aulacorhynchus* were constrained to be monophyletic. We used a Yule speciation process tree prior, running the program for 50×10^6^ generations, and sampling every 5000 generations. Convergence of chains to stationary was confirmed by inspection of posterior density of parameters in Tracer [44] and topological convergence in AWTY [45]. After discarding 2000 trees as burn-in, a maximum clade probability tree was obtained using the remaining 8000 trees.

## Results

Phylogenetic trees derived from ML and Bayesian analyses, based on the concatenated matrix of mitochondrial and nuclear data, were highly congruent, and showed significant nodal support for major clades. Given these results, we proceeded to analyze biogeographic information in the context of phylogenetic inference.

### Hypothesis Testing and Reconstruction of Ancestral Areas

None of the topologies consistent with alternate biogeographic hypotheses was found among the set of Bayesian post-burn-in trees. This result indicates that the Bayesian posterior probability of these topologies is close to zero (contingent on the model, data, prior probabilities, and convergence of the MCMC [37]).

**Figure 3:**
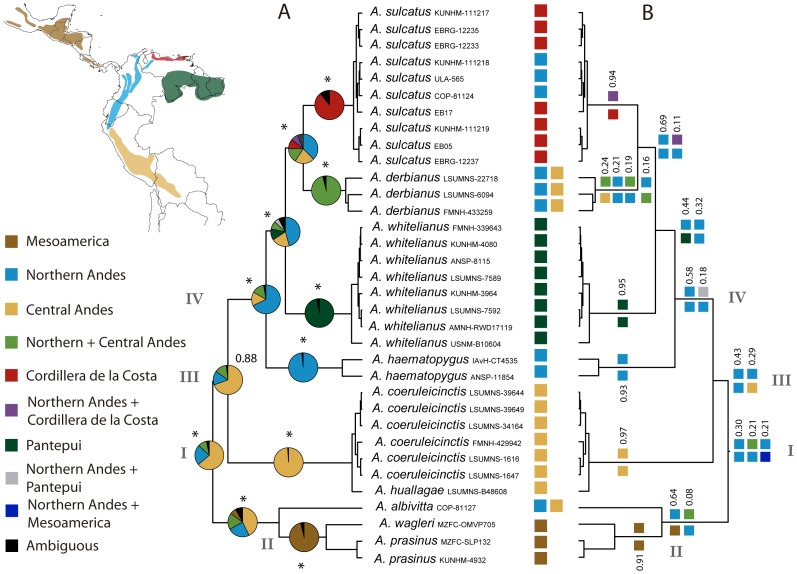
Ancestral area reconstruction for species in the genus *Aulacorhynchus*. Colors indicate geographic areas and combinations of up to two areas, and roman numerals indicate nodes of interest. A: Bayesian Binary MCMC Analysis in RASP; pie charts indicate the marginal probability of each area at nodes of interest; nodal support expressed as Bayesian posterior probabilities are showed above pie charts, with asterisks indicating Bayesian posterior probabilities = 1.00. B: Dispersal-Extinction-Cladogenesis analysis in Lagrange; numbers above splits indicate their relative probability; for simplicity, only splits summing ≥70 relative probability are shown.

The Bayesian binary MCMC analysis in RASP and the likelihood DEC analysis in Lagrange produced different results regarding the ancestral area of *Aulacorhynchus* (Figure 3). RASP, inferred the Central Andes as the most probable ancestral area for the genus (Node I, *P* = 0.64); other probable areas included the Northern Andes (*P* = 0.23) and a combination of the Central and Northern Andes (*P* = 0.1). Also, the Central Andes was the most probable ancestral area reconstructed at Nodes II (*P* = 0.43) and III (*P* = 0.7), followed by the Northern Andes, and a combination of both areas.

Lagrange, on the other hand, showed a first split in which the Northern Andes is the most probable ancestral state for *Aulacorhynchus* (Node I, *P* = 0.3), followed by two equally probable splits (*P* = 0.2): (1) Central Andes+Northern Andes, and Northern Andes; (2) Northern Andes, and Northern Andes+Mesoamerica. Also, at Nodes II and III an ancestral origin in the Northern Andes is always more probable than an origin in the Central Andes.

RASP and Lagrange showed the Northern Andes as the most probable ancestral state for Node IV. Also, both analyses showed that species distributed in Cordillera de la Costa and the Pantepui, originated from dispersal of an ancestor from the Northern Andes. Presence of *A. sulcatus* in the Northern Andes is most likely explained by secondary dispersal from Cordillera de la Costa (RASP) or persistence in an area composed by the Northern Andes and Cordillera de la Costa (Lagrange).

### Hypothetic Time of Diversification in *Aulacorhynchus*

**Figure 4:**
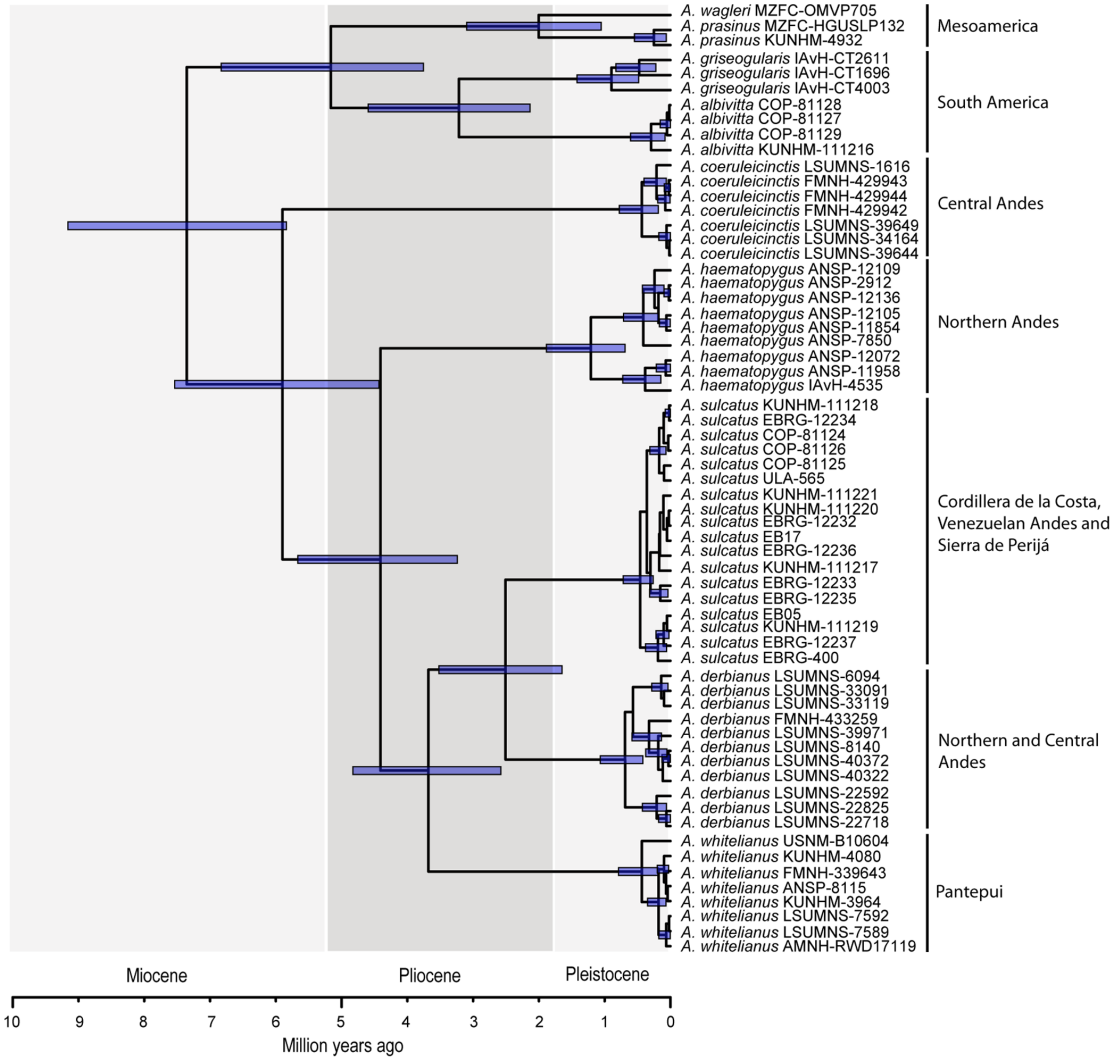
BEAST analysis showing the timeframe estimated for the evolutionary history of species in the genus *Aulacorhynchus*. Bars at each node indicate the highest posterior density (HPD) interval.

According to the BEAST analysis (Figure 4), and considering the highest posterior density (HPD) interval at each node, the origin of taxa in the genus *Aulacorhynchus* occurred between the late Miocene and the early Pleistocene (95% HPD = 9.2–1.6 Mya). The analysis indicates that all currently recognized species likely originated prior to the Pleistocene, with one exception: *A. sulcatus* and *A. derbianus* split at some time between the mid Pliocene and the early Pleistocene (95% HPD = 3.6–1.6 Mya). Interestingly, the divergence between the Mesoamerican and South American lineages of *A. prasinus* (95% HPD = 6.9–3.8 Mya) is estimated as being older than the origin of all currently recognized species except *A. coreuleicinctis*. In other *Aulacorhynchus* species, subspecific taxa originated mainly within the last 2 million years. The separation of the Pantepui lineage, *A. whitelianus*, from its sister clade, *A. derbianus*+*A. sulcatus* (Andes+Cordillera de la Costa), occurred within the Pliocene (95% HPD = 4.9–2.6 Mya).

## Discussion

### Biogeography of *Aulacorhynchus*

A hypothesis for the evolutionary origin of species in the genus *Aulacorhynchus* was first presented by Haffer [17] in his landmark work Avian Speciation in Tropical America. Therein, he proposed a sequential dispersal of an ancestral species from the Andes (*A. derbianus*) into the Pantepui (*A. whitelianus*), the eastern Cordillera de la Costa (*A. sulcatus erythrognathus*), the central Cordillera de la Costa (*A. s. sulcatus*), and west to the Venezuelan Andes and the mountains of northeast Colombia (*A. s. calorhynchus*). This sequence would have occurred during cool, glacial periods of the Pleistocene that lowered life zones across the humid montane forests. Haffer’s ideas were settled upon the prevailing taxonomy that suggested a common origin of the Andean and Pantepui populations of *A. derbianus*, and the observation of progressive changes in bill morphology (shape and coloration) in populations from contiguous but isolated montane areas. Molecular data showing that populations of *A. derbianus* from the Andes are more closely related to *A. sulcatus* than to *A. whitelianus* [32] indicate that Haffer’s proposal needs revision.

Topology tests showed that the phylogenetic arrangement found by Bonaccorso et al. [32] in which *A. derbianus* (Andes) and *A. sulcatus* (Cordillera de la Costa) are sister species, is significantly more likely than those implying a sister relationship between *A. whitelianus* and the remaining species in the genus, or a sister relationship between *A. whitelianus* and *A. sulcatus*. These results reject a biogeographic origin of *Aulacorhynchus* in the ‘ancient tepui’, as well as a close relationship between lineages from the Pantepui and Cordillera de la Costa.

Furthermore, ancestral area reconstructions showed an Andean origin for *Aulacorhynchus* (Figure 3). Whether the ancestral area of the genus is the Northern or the Central Andes, or both, is debatable. First, the RASP analysis (were the Central Andes had the highest probability of being the ancestral area) allowed dispersal among all biogegoraphic areas. In the Lagrange analysis (were the Northern Andes had the highest probability of being the ancestral area), we were able to restrict dispersal among non-adjacent areas. Thus, since RASP imposes no restrictions on dispersals, it allows inferring the Central Andes as an ancestral area in Node II. In RASP, this configuration in Node II has an important influence on the optimization of the Central Andes as the most likely ancestral area in Node I, which represents the ancestor of all *Aulacorhynchus*. This reconstruction is much less likely in Lagrange, because dispersal between Mesoamerica and the Central Andes was not allowed.

Second, the DEC optimization of ancestral states allows incorporating information from branch lengths to inform biogeographic inferences. This procedure avoids underestimating evolutionary change in terms of range expansions or contractions (dispersal vs. local extinction; [39]). Thus, in Lagrange, a proportionally long branch such as that conducting to *A. coeruleicinctis* (from the Central Andes), may have an important influence in optimizing other ancestral states over the Central Andes at a Node III, reducing the probability of optimizing the Central Andes at Node I.

Nevertheless, clear biogeographic patterns emerge within *Aulacorhynchus*. The Northern Andes has a higher probability of being the source of lineages that now inhabit the Cordillera de la Costa and the Pantepui (both analysis), and even the Central Andes (Lagrange). Most likely, it is also the source of the Mesoamerican lineage, since dispersal between the Central Andes and Mesoamerica is highly improbable. Our results are consistent with recent multi-species analyses in tanagers (Thraupini) indicating that the Northern Andes has been a source for lineages in other regions, with more dispersal events happening out of, than into this region [28].

On the other hand, examination of geographic patterns of *Aulacorhynchus* toucanets leaves an unresolved question. Considering that *A. derbianus* is distributed from Bolivia to northern Ecuador, and its sister species, *A. sulcatus*, is distributed from northwestern Colombia (Sierra de Perijá and Sierra Nevada de Santa Marta) to eastern Venezuela (Figure 1), what is the process explaining their absence from the Colombian Andes? Similar discontinuous distributions bridging the main Andes of Colombia are seen in other Andean birds such as *Grallaria haplonota*, *Turdus olivater* [50], and *Buthraupis montana* [51], among others. Whereas poor sampling across the region is still a possibility, in the case of the conspicuous *Aulacorhynchus* toucanets, we are inclined to support a scenario of local extinction. Whether the presence of *A. haematopygus* along the Andes of Colombia might have played a role in the potential competitive exclusion of a population of *A. derbianus* or *A. sulcatus* (or their ancestor) in this region, remains uncertain and difficult to test.

In the temporal realm, BEAST analyses indicated that diversification of *Aulacorhynchus* as a whole has been a process that started at ∼9.2–5.9 Mya, with all major lineages and most currently recognized species originating before the Pleistocene (Figure 4). Separation between *A. whitelianus* (Pantepui) and *A. sulcatus*+*A. derbianus* (Cordillera de la Costa+Andes) occurred sometime between 5.9 and 2.6 Mya, which negates the possibility of separation between Andean and Pantepui populations after the Pleistocene. Still, patterns of genetic differentiation seen in virtually all populations of *Aulacorhynchus* (except those in the *A. prasinus* complex) have a recent origin (∼2 Mya; Figure 4), and may have resulted from the sequential expansion and isolation of forest regions during the glacial cycles of the Quaternary. However, adequate testing of the processes behind this genetic pattern requires more in-depth population-level sampling and analyses.

Separation of South American and Mesoamerican ligeages of *Aulacorhyncus prasinus* occurred at ∼6.9–3.8 Mya. This estimate suggests that dispersal from South America northward into Mesoamerica may have taken place prior to the accepted time range estimate for the completion of the Isthmus of Panama ∼3.5–2.5 Mya [52]. However, the closure date of the isthmus is based on evolutionary divergence of marine organisms and therefore must be considered as a minimum age [53]. Other organisms might have dispersed across the Isthmus before the accepted 3.5–2.5 Mya estimate. In fact, exchange of vertebrate lineages between continents prior to the completion of the Isthmus has been documented for mammals [54], based on fossil data, and *Diglossa* flower piercers [27], *Campylorhynchus* wrens [55], some tanagers [56], *Pristimantis* frogs [57], and viperid snakes [58], among others, based on molecular data.

Biological exchange between North America and South America (or *vice-versa*) prior to the formation of the isthmus, may be explained by the existence of a string archipelago that connected both continents [59], [52] and allowed dispersal by island hopping. However, a recent study based on stratigraphic data suggests that southern Central America had coalesced into a peninsula connected to North America by 19 Mya [60]. Although this study does not provide a probable date for the closure of the isthmus, it seems that dispersal over a singular see channel would be more plausible than dispersal along a string archipelago. For *Aulacorhynchus* toucanets, as well as for other forest birds, movement across continents must have been constrained by the potential existence of suitable habitat along the peninsula and by their ability to disperse across the sea channel.

Regardless of interesting aspects that still need careful consideration and study, biogeographic analyses conducted on the phylogeny of *Aulacorhynchus* toucanets fit the predictions of a particular biogeographic hypothesis for the origin of Pantepui biotas. They support an origin in the Andes and posterior dispersal into the Pantepui, as first proposed by Mayr and Phelps [1], in a time window before the Pleistocene (contra Haffer [17]). However, we concede that our time estimates are only as good as the current methods for dating evolutionary events. Placing biogeographic processes in an evolutionary time framework across avian lineages may well have limitations in the calibration of evolutionary rates [48], [49], unexpected rate variation among lineages, and genetic polymorphism in ancestral populations [61].

### The Origin of Pantepui Diversity

Our results support previous analyses based on shared distributions of birds [1], that pointed to Andean origins of many subtropical Pantepui endemics. To our knowledge, only two phylogenetic analyses have included sufficiently dense species sampling to uncover the evolutionary origins of Pantepui montane lineages. The first, by Pérez-Emán [26], was a pioneering analysis of the Neotropical redstarts in the genus *Myioborus*. Therein, the Pantepui clade (*M. albifacies*, *M. cardonai*, and *M. castaneocapillus*) was sister to the endemic species from the Paria Peninsula of the Cordillera de la Costa (*M. pariae*). This result contrasts with ours in indicating close relationships between Pantepui and coastal elements. Unfortunately, further biogeographic interpretation regarding routes of colonization of *Myioborus* between these areas was precluded by low phylogenetic resolution at interior nodes [26].

The second study, by Mauck and Burns [27], focused on *Diglossa* flowerpiercers. In this study, species from the Pantepui (*D. major* and *D. duidae*) form a clade that is sister to species from the Andes and Cordillera de la Costa. They identified the Andes as the ancestral area for the genus, with Pantepui and Central American species founded via single colonization events. Although they assigned the species from Cordillera de la Costa (*D. venezuelensis*) to the Andean region, it seems clear from their tree that it would represent yet another independent dispersal event. Their results are similar to ours regarding areas of origin and direction of dispersal. Also, in a broader biogeographic reconstruction for tanagers, Sedano and Burns [28] identified the Northern Andes as one of the sources of Pantepui taxa, with a low probability for dispersal events from the Pantepui to the Andes.

The emerging picture of the origin of Pantepui biota is complex, with some taxa dispersing from the Pantepui (bromeliads [20], [21]), others dispersing from the Andes (flowerpiercers [27]; tanagers [28]; *Aulacorhynchus* toucanets, this paper), or having close affinities with the Cordillera de la Costa (redstarts [26]). Phylogenetic studies in frogs [62] and Rapateaceae plants [63] have identified the surrounding lowlands as yet another source of diversity. The question of why only some species reach and colonize the Pantepui may require a deeper and more precise understanding of species’ dispersal capacities, competitive abilities, niche breath, and adaptability to new conditions, among others.

### Conservation of Pantepui Populations

It is clear that morphological similarity must not preclude careful examination of isolated populations of the Pantepui region. Although diagnosable from one another, *Aulacorhynchus derbianus* and *A. whitelianus* are morphologically very similar, and yet, do not form a monophyletic lineage. The tepui redstart, *Myioborus castaneocapilla,* was formerly considered a northern population of the brown-capped redstart *M. brunniceps*, from western Argentina [26], [29]. Thus, likely, Pantepui populations of many other organisms are evolutionary significant units [64] and may represent endemic species level taxa as well. This consideration is important given recent phylogenetic studies suggesting high rates of homoplasy in Neotropical birds, caused by natural [65] and sexual selection [66], processes that may conceal good biological and evolutionary species.

Excepting the highlands in the Brazilian Shield, the Pantepui is the most isolated montane region in the Neotropics. Separated from the Andes and the Cordillera de la Costa by the extensive lowlands of the Amazon and Orinoco basins [13], the region possess incredible potential for speciation and genetic diversification. Moreover, biotic [14] and phylogenetic differences [26] between eastern and western tepui groups, imply additional within-Pantepui divergence. Considering the high potential for cryptic speciation and genetic isolation of Pantepui populations, phylogenetic studies of these taxa are important from a conservation perspective. This information is particularly relevant when new taxonomic assessments may indicate a reduction in geographic range (or Extent of Occurrence), which is one of the criteria determining species’ conservation status [67].

## Supporting Information

Table S1 **List of tissue samples and GenBank accession numbers for sequences of species included in the present study.**
(PDF)
