# Supplementary material for: On the Origin of Pantepui montane biotas: A Perspective Based on the Phylogeny of Aulacorhynchus toucanets
Source: PLoS One. 2013 Jun 26;8(6):e67321. doi: 10.1371/journal.pone.0067321 (PMC3694146; doi:10.1371/journal.pone.0067321)
Supplement: Table S1 — List of tissue samples and GenBank accession numbers for sequences of species included in the present study. (PDF) [file pone.0067321.s001.pdf]

Table S1. List of tissue samples and GenBank accession numbers for sequences of species included in the present study. Acronyms: AMNH, American Museum of Natural History; ANSP, Academy of Natural Sciences; COP, Colección Ornitológica Phelps, Venezuela; EBRG, Museo Estación Biológica Rancho Grande, Venezuela; FMNH, Field Museum of Natural History; IAvH, Instituto Alexander von Humboldt, Colombia; KUNHM, University of Kansas Natural History Museum; LSUMNS, Louisiana State University Museum of Natural Science; MZFC, Museo de Zoología, Facultad de Ciencias, Universidad Nacional Autónoma de México; ULA, Universidad de los Andes, Venezuela; USNM, National Museum of Natural History, Smithsonian Institution; EB = field number (Elisa Bonaccorso), no voucher associated. *Sequences from Weckstein 2005. Taxonomy follows Remsen *et al.* (2012) and Bonaccorso *et al.* (2011).

**Taxa**	**Museum and tissue number**	**Locality Information**	**ND2**	**cyt*b***	**βfib7**	**TGFβ2.5**
A. prasinus	MZFC-HGUSLP132	Mexico: Hidalgo; Pisaflores, El Coyol	JF424372	JF424451	JF424519	JF424557
A. prasinus	KUNHM-4932	El Salvador: Morazán; Cerro Cacahuatique	JF424374	JF424453	JF424521	JF424559
A. wagleri	MZFC-OMVP705	Mexico: Oaxaca; Putla, Sta Ana del Progreso	JF424373	JF424452	JF424520	JF424558
A. albivitta	COP-81127	Venezuela: Zulia; Sierra de las Lajas, Serranía de Perijá	JF424376	JF424454	JF424522	JF424560
A. albivitta	COP-81128	Venezuela: Zulia; Sierra de las Lajas, Serranía de Perijá	—	JF424455	—	—
A. albivitta	COP-81129	Venezuela: Zulia; Sierra de las Lajas, Serranía de Perijá	—	JF424456	—	—
A. albivitta	KUNHM-111216	Venezuela: Mérida; La Mucuy, P. N. Sierra Nevada	—	JF424457	—	—
A. griseogularis	IAvH-CT2611	Colombia: Caldas; El Laurel, Aranzazu	—	JF424458	—	—
A. griseogularis	IAvH-CT1696	Colombia: Rizalda; La Cumbre, Pueblo Rico	—	JF424459	—	—
A. griseogularis	IAvH-CT4003	Colombia: Valle del Cauca; Chicoral, La Cumbre	—	JF424460	—	—
A. coeruleicinctis	LSUMNS-1616	Peru: Pasco; Santa Cruz, about 9 km SSE Oxapampa	JF424439	JF424508	JF424547	JF424587
A. coeruleicinctis	FMNH-429942	Peru: Cuzco; Pillahuata	JF424441	JF424510	JF424549	JF424589
A. coeruleicinctis	FMNH-429943	Peru: Cuzco; Pillahuata	—	JF424511	—	—
A. coeruleicinctis	FMNH-429944	Peru: Cuzco; Pillahuata	—	JF424512	—	—
A. coeruleicinctis	LSUMNS-34164	Bolivia: Santa Cruz; Chuchial, Ca 37 km, SE Samaipata	JF424445	JF424513	JF424550	JF424590
A. coeruleicinctis	LSUMNS-39644	Bolivia: Santa Cruz; La Pajcha, Ca 28 km S Samaipata	JF424446	JF424514	JF424551	JF424591
A. coeruleicinctis	LSUMNS-39649	Bolivia: Santa Cruz; La Pajcha, Ca 37 km S Samaipata	JF424447	JF424515	JF424552	JF424592
A. huallagae	LSUMNS-B48608	Peru: La Libertad; Cumpang	JF424438	—		—
A. haematopygus sexnotatus	IAvH-CT 4535	Colombia: Antioquia; Anori, El Llano	JF424427	JF424499	JF424545	JF424585
A. haematopygus sexnotatus	ANSP-11854	Ecuador: Esmeraldas; El Placer	JF424428	JF424500	JF424546	JF424586
A. haematopygus sexnotatus	ANSP-11958	Ecuador: Esmeraldas; El Placer	—	JF424501	—	—

A. haematopygus sexnotatus	ANSP-12105	Ecuador: Pichincha; Mindo	—	JF424502	—	—
A. haematopygus sexnotatus	ANSP-12109	Ecuador: Pichincha; Mindo	—	JF424503	—	—
A. haematopygus sexnotatus	ANSP-12136	Ecuador: Pichincha; Mindo	—	JF424504	—	—
A. haematopygus sexnotatus	ANSP-12072	Ecuador: Pichincha; Mindo	—	JF424505	—	—
A. haematopygus sexnotatus	ANSP-2912	Ecuador: El Oro; Machalilla, Cerro San Sebastián	—	JF424506	—	—
A. haematopygus sexnotatus	ANSP-7850	Ecuador: El Oro; 9.5 km W Piñas	—	JF424507	—	—
A. whitelianus duidae	LSUMNS-7589	Venezuela: Amazonas; Cerro Neblina	JF424414	JF424490	JF424536	JF424576
A. whitelianus duidae	LSUMNS-7592	Venezuela: Amazonas; Cerro Neblina	JF424415	JF424491	JF424537	JF424577
A. whitelianus duidae	AMNH-RWD 17119	Venezuela: Amazonas; Río Mawarinumo	JF424416	JF424492	JF424538	JF424578
A. whitelianus whitelianus	FMNH-339643	Venezuela: Bolívar; Santa Elena Highway, Km 118	JF424417	JF424493	JF424539	JF424579
A. whitelianus whitelianus	ANSP-8115	Guyana: Potaro-Siparuni; Iwokrama Mountains	JF424420	JF424494	JF424540	JF424580
A. whitelianus osgoodi	KUNHM-3964	Guyana: East Berbice-Corentyne; Acari Mountains, N side	JF424421	JF424495	JF424541	JF424581
A. whitelianus osgoodi	KUNHM-4080	Guyana: East Berbice-Corentyne; Acari Mountains, N side	JF424422	JF424496	JF424542	JF424582
A. whitelianus osgoodi	USNM-B10604	Guyana: East Berbice-Corentyne; Acari Mountains, N side	JF424423	JF424497	JF424543	JF424583
A. derbianus derbianus	LSUMNS-6094	Ecuador: Morona Santiago; Cordillera del Cutucú	JF424403	JF424479	JF424533	JF424573
A. derbianus derbianus	LSUMNS-33091	Peru: Cajamarca; Ca 3km NNE San José de Lourdes	—	JF424480	—	—
A. derbianus derbianus	LSUMNS-33119	Peru: Cajamarca; Ca 3km NNE San José de Lourdes	—	JF424481	—	—
A. derbianus derbianus	LSUMNS-39971	Peru: Loreto; Ca. 86 km SE Juanjui on E bank upper Río Pauya	—	JF424482	—	—
A. derbianus derbianus	LSUMNS-40372	Peru: Loreto; Ca. 86 km SE Juanjui on E bank upper Río Pauya	—	JF424483	—	—
A. derbianus derbianus	LSUMNS-40322	Peru: Loreto; Ca. 86 km SE Juanjui on E bank upper Río Pauya	—	JF424484	—	—
A. derbianus derbianus	LSUMNS-8140	Peru: Pasco; Cushi	—	JF424485	—	—
A. derbianus derbianus	FMNH-433259	Peru: Cuzco; Consuelo, 15.9 km SW Pilcopata	JF424410	JF424486	JF424534	JF424574
A. derbianus	LSUMNS-22592	Bolivia: La Paz, 83 km by road E Charazani,	—	JF424487	—	—

derbianus		Cerro Asunta Pata				
A. derbianus derbianus	LSUMNS-22718	Bolivia: La Paz, 83 km by road E Charazani, Cerro Asunta Pata	JF424412	JF424488	JF424535	JF424575
A. derbianus derbianus	LSUMNS-22825	Bolivia: La Paz, 83 km by road E Charazani, Cerro Asunta Pata	—	JF424489	—	—
A. sulcatus sulcatus	KUNHM-111219	Venezuela: Aragua; Estación Biológica Rancho Grande	JF424385	JF424461	JF424523	JF424563
A. sulcatus sulcatus	EB05-No voucher	Venezuela: Aragua; Estación Biológica Rancho Grande	JF424386	JF424462	JF424524	JF424564
A. sulcatus sulcatus	EBGR-12237	Venezuela: Aragua; Estación Biológica Rancho Grande	JF424387	JF424463	JF424525	JF424565
A. sulcatus sulcatus	EBGR-400	Venezuela: Aragua; Estación Biológica Rancho Grande	—	JF424464	—	—
A. sulcatus calorhynchus	KUNHM-111218	Venezuela: Lara; El Hacha, Parque Nacional Yacambú	JF424389	JF424465	JF424526	JF424566
A. sulcatus calorhynchus	EBGR-12234	Venezuela: Lara; El Hacha, Parque Nacional Yacambú	—	JF424466	—	—
A. sulcatus calorhynchus	ULA-565	Venezuela: Mérida; San Luís	JF424391	JF424467	JF424527	JF424567
A. sulcatus calorhynchus	COP-81124	Venezuela: Zulia; Sierra de las Lajas, Serranía de Perijá	JF424392	JF424468	JF424528	JF424568
A. sulcatus calorhynchus	COP-81125	Venezuela: Zulia; Sierra de las Lajas, Serranía de Perijá	—	JF424469	—	—
A. sulcatus calorhynchus	COP-81126	Venezuela: Zulia; Sierra de las Lajas, Serranía de Perijá	—	JF424470	—	—
A. sulcatus sulcatus × *calorhynchus?*	KUNHM-111221	Venezuela: Guárico; Hacienda Picachito, Cerro Platillón	—	JF424471	—	—
A. sulcatus sulcatus × *calorhynchus?*	EBGR-12233	Venezuela: Guárico; Hacienda Picachito, Cerro Platillón	JF424396	JF424472	JF424529	JF424569
A. sulcatus erythrognathus	KUNHM-111217	Venezuela: Sucre; Las Melenas, Península de Paria	JF424397	JF424473	JF424530	JF424570
A. sulcatus erythrognathus	EBGR-12235	Venezuela: Sucre; Las Melenas, Península de Paria	JF424398	JF424474	JF424531	JF424571
A. sulcatus erythrognathus	EBGR-12236	Venezuela: Sucre; Las Melenas, Península de Paria	—	JF424475	—	—
A. sulcatus erythrognathus	KUNHM-111220	Venezuela; Sucre; La Medianía	—	JF424476	—	—
A. sulcatus erythrognathus	EB17-no voucher	Venezuela; Sucre; La Medianía	JF424401	JF424477	JF424532	JF424572
A. sulcatus erythrognathus	EBGR-12232	Venezuela; Sucre; La Medianía	—	JF424478	—	—
Outgroup						

Andigena cucullata	LSUMNS-1273	Bolivia: La Paz	AY959855*	AY959828*	—	—
Andigena hypoglauca	FMNH-433261	Peru: Cuzco; La Esperanza, 39 km (road) NE Paucartambo	—	—	JF424553	JF424593
Selenidera gouldii	FMNH-389772	Brazil: Rondonia; Cachoeira Nazare, W bank Rio Jiparana	JF424448	JF424516	JF424554	JF424594
Pteroglossus azara	KUNHM-749	Peru: Madre de Dios	JF424449	JF424517	JF424555	JF424595
Ramphastos sulfuratus	KUNHM-2060	Mexico: Campeche	JF424450	JF424518	JF424556	JF424596
